# The spectrogram data of quinazoline derivatives containing a dithioacetal moiety

**DOI:** 10.1016/j.dib.2018.08.085

**Published:** 2018-09-12

**Authors:** Dandan Xie, Jing Shi, Awei Zhang, Zhiwei Lei, Guangcheng Zu, Yun Fu, Xiuhai Gan, Limin Yin, Baoan Song, Deyu Hu

**Affiliations:** State Key Laboratory Breeding Base of Green Pesticide and Agricultural Bioengineering, Key Laboratory of Green Pesticide and Agricultural Bioengineering, Ministry of Education, Guizhou University, Huaxi District, Guiyang 550025, China

## Abstract

The nuclear magnetic resonance, and high-resolution mass spectrometry of quinazoline derivatives containing a dithioacetal moiety, which was hosted in the research article entitled “Syntheses, antiviral activities and induced resistance mechanisms of novel quinazoline derivatives containing a dithioacetal moiety”. The data include ^1^H nuclear magnetic resonance (^1^H NMR), ^13^C nuclear magnetic resonance (^13^C NMR), and high-resolution mass spectrometry. In this article, a more comprehensive data interpretation and analysis is explained.

**Specifications table**

**Table:** 

Subject area	Organic chemistry
More specific subject area	pesticide
Type of data	figures
How data was acquired	The nuclear magnetic resonance spectra were acquired by JEOL-ECX500 MHz (JEOL, Tokyo, Japan) or Bruker DPX 400 MHz (Bruker BioSpin GmbH, Rheinstetten, Germany). The high-resolution mass spectrometry was acquired through Thermo Scientific Q Exactive (Thermo Fisher Scientific, Massachusetts, America).
Data format	Analyzed
Experimental factors	The quinazoline derivatives were synthesized and purified *via* chemistry route. The ^1^H NMR and ^13^C NMR were acquired by instruments with CDCl_3_ or dimethyl sulfoxide (DMSO) as the solvent with tetramethylsilane (TMS) as an internal standard, and chemical shifts are expressed in *δ* (ppm).
Experimental features	Through the sharing of nuclear magnetic resonance and high-resolution mass spectrometry, the chemical structure of the compounds can be determined.
Data source location	Guiyang city, China
Data accessibility	The data are included with this article

**Value of the data**

• The data confirmed the correct structure of these first time synthesized compounds **4a**-**4x**.
• The data as a background for the bioassay and quantitative structure-activity relationship analysis of compounds **4a**-**4x**.
• The data severs as a benchmark for other researchers synthesize this type of compound in the future.

## 1 Data

**Fig. 1:**
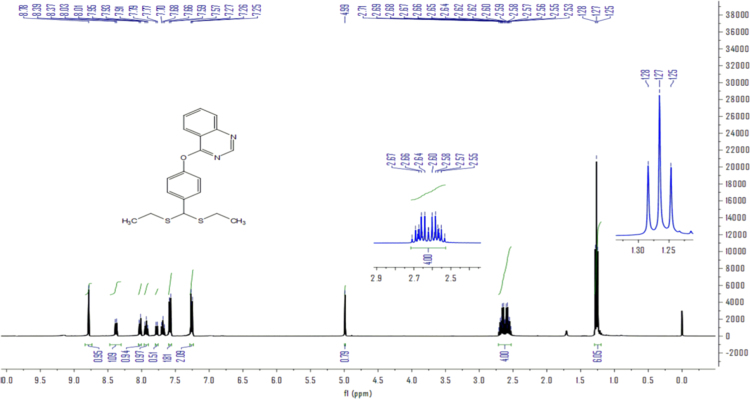
^1^H NMR of Compound **4a**.

**Fig. 2:**
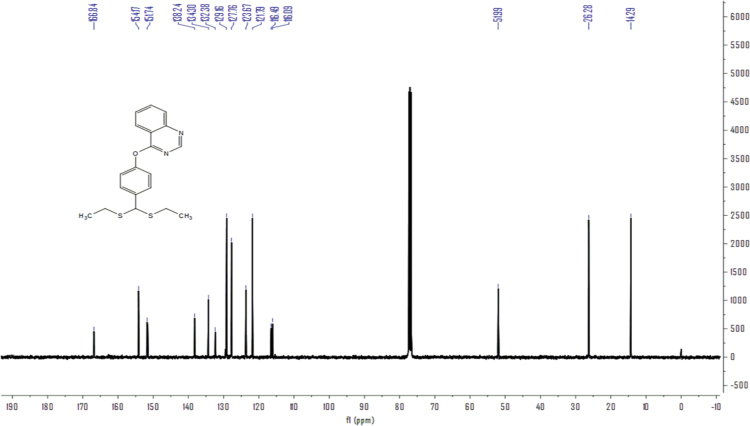
^13^C NMR of Compound **4a**.

**Fig. 3:**
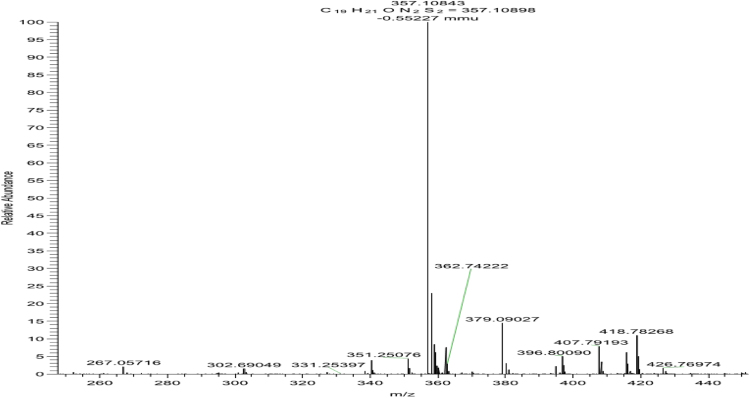
HRMS of Compound **4a**.

The dataset of this article provide information on the spectra of 22 quinazoline derivatives contain a dithioacetal moiety. The ^1^HNMR, ^13^CNMR and HRMS spectra of compound **4a** were shown in Fig. 1, Fig. 2, Fig. 3, respectively.

## 2 Experimental design, materials and methods

The sample was dissolved in the CDCl_3_ or DMSO-*d*_*6*_ to prepare a solution with a concentration of 5 mmol/L, the ^1^H NMR and ^13^C NMR were acquired by instruments with tetramethylsilane (TMS) as an internal standard at room temperature [1], [2]. The initial data obtained were analyzed by the *Mestrenova 6.2.0* software and the chemical shifts and integral areas of each hydrogen atom and carbon atom were obtained.

A trace sample is dissolved in guaranteed methanol and then tested by a Thermo Scientific Q Exactive mass spectrograph. The initial data obtained were analyzed by the Exactive Series 2.4 software.
